# Breaking the Cycle: Addressing Period Poverty as a Critical Public Health Challenge and Its Relation to Sustainable Development Goals

**DOI:** 10.7759/cureus.62499

**Published:** 2024-06-16

**Authors:** Nor Faiza Mohd. Tohit, Mainul Haque

**Affiliations:** 1 Department of Community Health, Universiti Pertahanan Nasional Malaysia (National Defence University of Malaysia), Kuala Lumpur, MYS; 2 Department of Research, Karnavati Scientific Research Center (KSRC) School of Dentistry, Karnavati University, Gandhinagar, IND; 3 Department of Pharmacology and Therapeutics, National Defence University of Malaysia, Kuala Lumpur, MYS

**Keywords:** mental health, puberty, social equity, menstrual hygiene management, taboo, stigma, gender equality, menstrual health, adolescents, menstruation

## Abstract

This narrative review comprehensively examines the intricate relationship between period poverty and the Sustainable Development Goals (SDGs), positioning it as a critical public health challenge with far-reaching implications. Through an in-depth analysis of the multifaceted impact of period poverty on public health, including its effects on reproductive health, mental well-being, and economic participation, the paper underscores the urgent need to address this issue within the framework of the SDGs. An overview of existing literature on period poverty, its impact on health and well-being, and its relation to the SDGs was carried out. Different perspectives, interventions, and policy approaches to addressing period poverty were also explored. By illuminating the interplay between period poverty and various SDGs, particularly those related to gender equality, health, education, and economic empowerment, the study emphasizes the imperative of integrating menstrual health and hygiene into global development efforts. Advocating for targeted policies, funding, and advocacy, the manuscript calls for a holistic and inclusive approach to breaking the cycle of period poverty, ultimately contributing to advancing the SDGs and fostering a more equitable and healthier global society. Efforts to eradicate period poverty - providing affordable menstrual products, improving sanitation infrastructure, enhancing education, and implementing supportive policies - lead to significant progress in public health and gender equity. By prioritizing menstrual health management in public health policies, educational programs, and economic strategies, we can ensure that everyone who menstruates can do so with dignity and without limits on their potential.

## Introduction and background

Between puberty and menopause, adolescent girls and women experience around 459 menstrual cycles, averaging about 6.25 years of managing menstruation [1]. This results in a considerable monthly requirement for supportive social and physical settings to handle menstruation effectively. Adolescence, particularly with the onset of menstruation, represents a pivotal period in one's life [2]; exposure to various physical [3] and psychological [4] risks affects long-term health and well-being. Dealing with menstruation in challenging environments during adolescence can heighten girls' susceptibility to sexual violence, stigma, and discrimination, impacting their future health and well-being [4,5]. 

Period poverty is a pervasive public health crisis that spans the globe, affecting millions of women, girls, and people who menstruate [6]. It refers to inadequate access to menstrual hygiene products, education, and facilities due to financial constraints, stigmatization, and infrastructural deficiencies [6,7]. Period poverty is connected to the Sustainable Development Goals (SDGs) due to its impact on multiple goal areas [8]. The Sustainable Development Goals are a collection of 17 global goals set by the United Nations General Assembly in 2015 as part of the 2030 Agenda for Sustainable Development. It is a universal call to action to end poverty, protect the planet, and ensure that all people enjoy peace and prosperity. The SDGs are designed to be a blueprint to achieve a better and more sustainable future for all. They serve as a framework for countries and organizations to guide their policies and actions toward sustainable development [8,9]. The findings of this study hold significant implications for public health and sustainable development initiatives. First, this review underscores the pressing need for targeted interventions that address period poverty as a critical determinant of individuals' health and well-being [6]. The impact of inadequate menstrual hygiene management goes beyond mere hygiene, influencing health outcomes, educational attainment, and economic participation [2-4], all interconnected with broader public health goals. Addressing period poverty is thus fundamental to promoting good health and well-being, aligning with Sustainable Development Goal 3 [8]. Moreover, this study is intended to reveal that period poverty intersects with gender equality, highlighting the imperative for inclusive and gender-sensitive policies and programs to achieve Sustainable Development Goal 5 [8,9].

In the realm of sustainable development, the study emphasizes how period poverty undermines efforts to promote inclusive and equitable societies. Sustainable Development Goals cannot be fully realized without addressing the fundamental needs of marginalized communities affected by period poverty [10]. By recognizing the far-reaching implications of period poverty on public health and sustainable development, policymakers, practitioners, and advocates can foster more holistic and integrated approaches to build healthier and more resilient communities, ultimately advancing the global agenda for sustainable development [10,11]. Growing recognition of the profound implications of period poverty on individual well-being and broader societal health has underscored the urgent need for systemic changes. Period poverty intersects with various Sustainable Development Goals, profoundly impacting gender equality, health, education, and economic empowerment. Addressing period poverty is crucial for achieving the SDGs, as it directly affects women's and girls' rights and well-being, making it an integral component of global development efforts [8-11].

Actions aimed at eradicating period poverty - including providing access to affordable menstrual products, improving sanitation infrastructure, enhancing educational efforts, and implementing supportive policies - can lead to considerable advancements in public health and gender equity. By prioritizing menstrual health management in public health policies, educational programs, and economic strategies, we can ensure that everyone who menstruates can do so with dignity and without impediment to their potential [5-7].

## Review

Materials and methods

This comprehensive review aimed to analyze the existing body of literature on period poverty and its interconnectedness with public health challenges and the SDGs, consequently providing a holistic understanding of this critical public health issue. The review process involved a systematic search using academic databases such as Google Scholar and PubMed. The following keywords were employed to identify relevant literature: “period poverty,” AND “menstrual hygiene,” AND “public health,” AND “Sustainable Development Goals (SDGs).” The inclusion criteria prioritized articles, reports, and studies focusing on the intersection of period poverty and its impact on public health and development within the context of the SDGs. Both qualitative and quantitative studies, as well as gray literature, were considered during the review process.

The materials used in this review encompassed a wide range of scholarly articles, reports, and relevant documents obtained from reputable sources. The selection of materials included peer-reviewed articles, government reports, NGO publications, and international organization analyses. Additionally, these materials were selected based on their relevance to examining the multifaceted dimensions of period poverty and their implications for achieving the SDGs. The articles included are from the past 10 years, from all countries.

**Figure 1:**
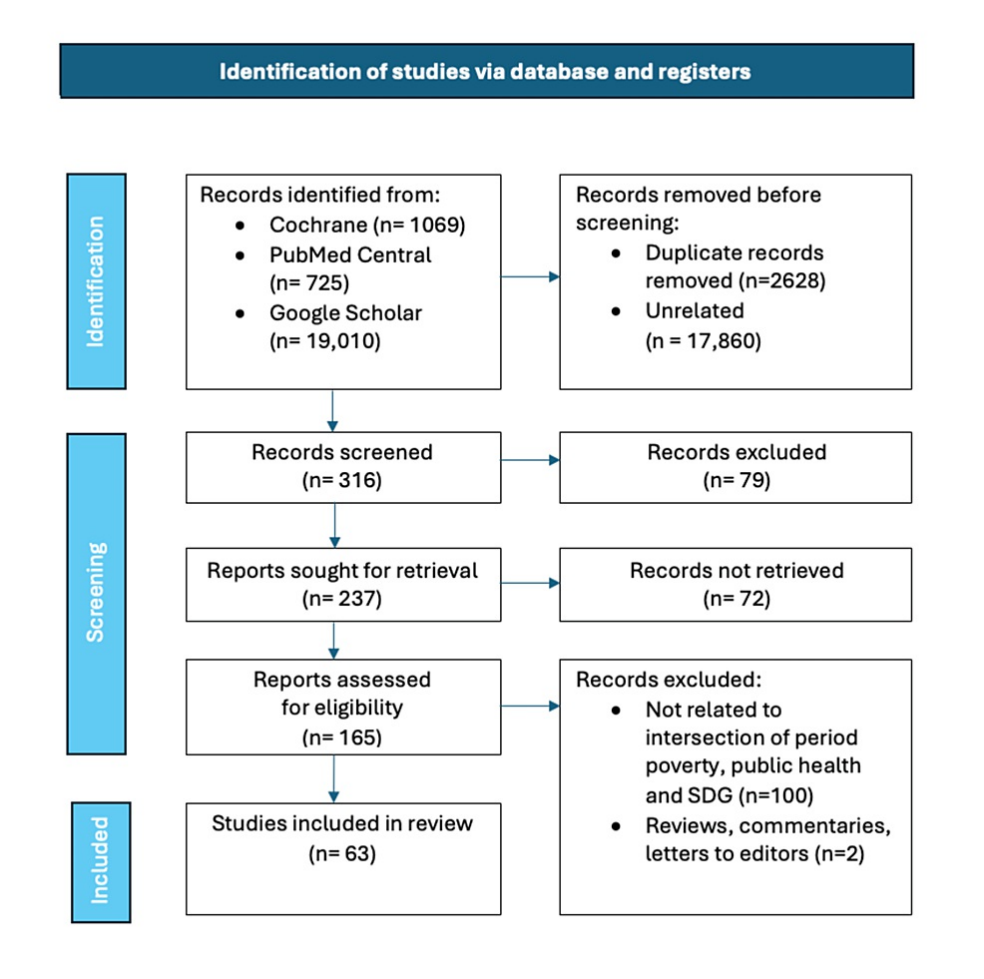
Illustrates the materials and methods of this review paper.

Articles excluded from the review encompassed those that did not directly relate to the intersection of period poverty, public health, and its connection to the Sustainable Development Goals. Additionally, any non-peer-reviewed or non-reputable sources and articles not available in English were excluded from consideration. Reviews, commentaries, and letters to the editor were excluded. The retrieved articles were screened. The focus remained on ensuring the inclusion of high-quality, relevant scholarly literature to address the specific theme of the review. Potential biases in a review article, such as selection bias, publication bias, and confirmation bias, can impact the integrity and objectivity of the review process. To counter these biases, the authors employed comprehensive search strategies, including diverse sources and seeking out unpublished studies, to help minimize selection and publication biases. Furthermore, using structured review methodologies and ensuring transparency in the decision-making process can mitigate confirmation bias. Both authors independently selected the articles by adhering to systematic and transparent approaches. In the event of disputes, the authors managed the disputes regarding including and excluding articles through structured discussions and consensus-building processes. This could involve detailing the methodology for article selection, explaining the criteria used for inclusion, and describing any conflict resolution mechanisms employed. The following flow chart (Figure 1) illustrates the materials and methods of this narrative review.

The impacts of period poverty on public health

**Figure 2:**
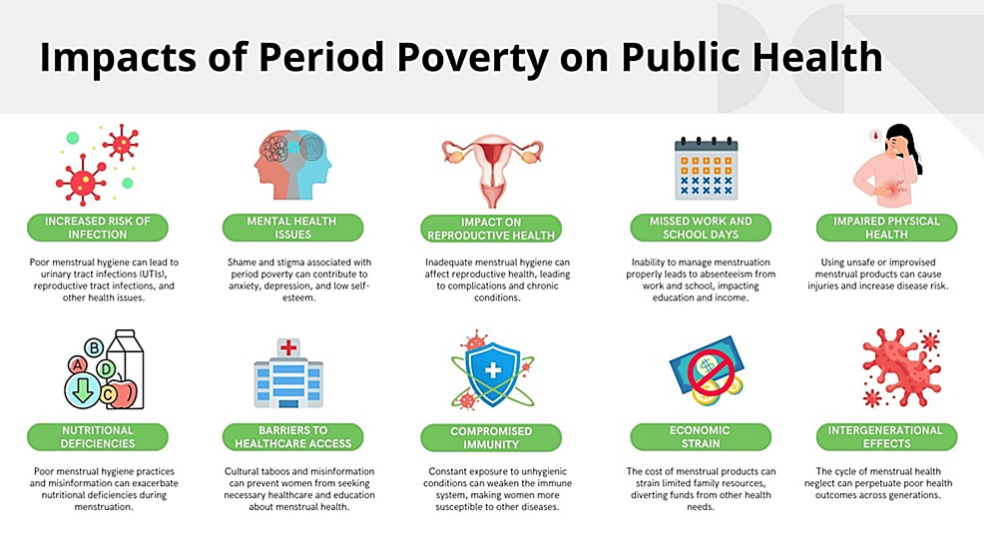
Impacts of period poverty on public health. Image credit: Nor Faiza Mohd. Tohit.

**Figure 3:**
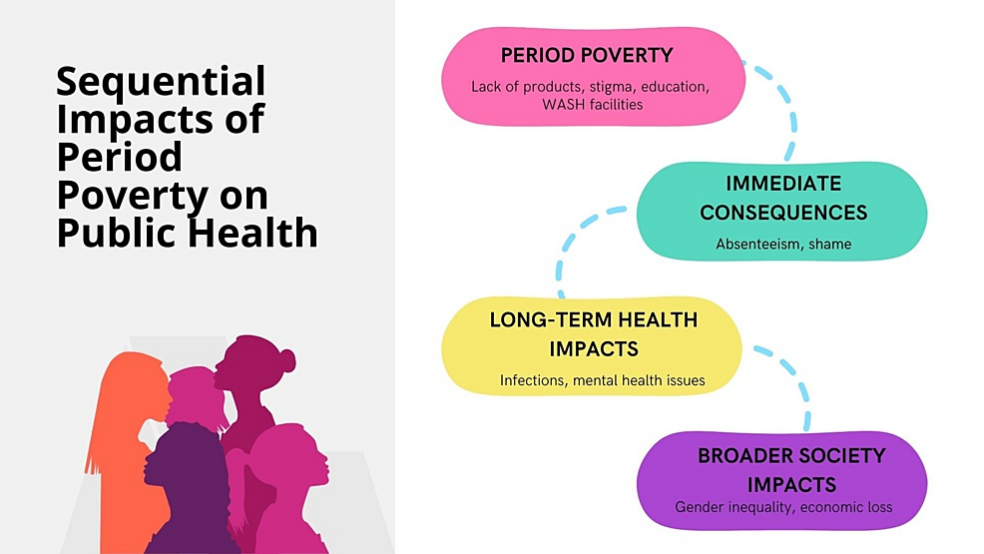
Sequential impacts of period poverty on public health. Image Credit: Nor Faiza Mohd. Tohit.

Period poverty is a pressing public health concern that affects millions of women and girls globally. This issue highlights significant disparities in health and well-being and intersects with broader social, economic, and educational inequalities. Figure 2 depicts the impacts of period poverty on public health, whereas Figure 3 illustrates the sequential implications for public health.

The market for feminine hygiene products generates billions of dollars annually, with menstruators spending approximately $3,000 to $5,000 on over 16,000 products throughout their lifetimes [12]. Financial obstacles often hinder menstruators, primarily self-identified women, from obtaining safe and healthy menstrual hygiene products. Consequently, many women cannot afford feminine hygiene products or make difficult choices between purchasing food or essential hygiene items [13].

At its core, period poverty fundamentally impacts health through increased exposure to infections and potentially severe medical conditions. The inability to afford suitable menstrual products forces many to resort to substitutes such as rags, paper, or even hay, posing significant health risks [14]. For instance, in a study conducted in Uganda, it was found that a substantial number of young girls experienced recurrent urogenital infections due to the use of unhygienic alternatives [15]. Such conditions not only affect immediate health outcomes but can lead to more chronic issues and even reproductive health problems, which highlight the severe implications of period poverty. Period poverty also significantly contributes to educational disparities, perpetuating a cycle of inequality and poverty. Across numerous developing countries, menstruation-related absenteeism is a prevalent concern [16]. The absence of adequate menstrual products and facilities discourages school attendance during menstrual periods. In Kenya, reports indicate that many girls miss approximately four school days each month, or almost 20% of a school year [17].

In Ohio, USA, research reveals the prevalence of period poverty among young individuals [18]. The participants responded to a survey evaluating their availability of menstrual products, their comprehension of sexual and menstrual health, their emotions regarding menstruation, and the perceived effects of their menstrual cycles on daily life. Due to limited access to hygiene products, students indicated using alternative items instead of pads or tampons, missing school or work, and harboring negative feelings toward their menstruation. Additionally, participants reported frequent absences from sports, work, social interactions with family and friends, and theater or music practices due to their periods.

In India, cultural stigmas and inadequate sanitation facilities result in a high dropout rate among adolescent girls, which perpetuates educational and economic disparities [19]. Out of 2332 schoolgirls in Bangladesh who had begun menstruating, 41% (931) reported missing school, averaging 2.8 days missed per menstrual cycle. Those who felt uncomfortable at school during their periods (99% vs. 32%; APD = 58%; CI: 54 to 63) and those who thought menstrual issues affected their school performance (64% vs. 30%; APD = 27; CI: 20 to 33) were more prone to school absence during menstruation. Additionally, girls who were restricted from any activities during menstruation were more frequently absent (41% vs. 33%; APD = 9.1; CI: 3.3 to 14) [20].

The interruption in education affects academic performance and has long-term economic implications, limiting future opportunities and contributing to poverty. A survey conducted among high school students in St. Louis, Missouri, examined period product insecurity, school absenteeism, and the use of school resources to access period products, revealing concerning insights into period poverty. Out of 119 respondents, nearly 64.4% (95% CI: 55.1-73.0%) indicated they experienced period product insecurity. Additionally, 66.9% (95% CI: 57.7-75.3%) mentioned using at least one of the school's resources to obtain period products. Moreover, 33.6% (95% CI: 25.0-43.1%) of the students reported missing school due to a shortage of period products [16].

Malaysia is not immune to the challenges posed by period poverty. Despite being a relatively developed nation, period poverty persists, particularly among low-income households and marginalized communities. A study by Plan International Malaysia 2021 revealed that nearly half (46%) of young girls in rural areas do not have adequate access to sanitary products [21]. This lack of access has profound implications for their education and overall well-being, with many reporting absenteeism from school during their menstrual cycles. Additionally, cultural and social stigmas surrounding menstruation remain prevalent, contributing to misinformation and shame, which further exacerbate the issue. Efforts by various non-governmental organizations (NGOs), such as the Malaysian Red Crescent Society's #myPADS program, seek to address these challenges by providing sanitary products and educational programs. However, systematic policy changes and comprehensive public health strategies are critical to effectively combating period poverty in Malaysia.

Menstruation, entrenched in intricate belief systems, is surrounded by numerous myths, taboos, and stigmatizing, negative, and shameful feelings. Many people perceive menstruation as dirty, messy, and impure, resulting in the notion that it is disgraceful and ought to be concealed [22]. As a collective, society views menstruating women as physically impaired during their menstrual phase or mentally imbalanced during the premenstrual phase. They are often seen as uncontrollable, unwell, irrational, and lacking femininity [23]. Consequently, girls and others who menstruate may feel isolated or be expected to handle menstruation privately to avoid what is seen as unpleasant or as a form of physical and spiritual contamination.

The intensity of menstrual stigma experiences varies by context, but such stigma is pervasive, long-standing, and deeply rooted. This stigma, whether internalized by those who menstruate or expressed by others, along with related discriminatory practices, has significant adverse effects on the well-being of girls, women, and others who menstruate, impacting their freedom, mobility, dignity, and health [24]. Period poverty not only affects physical health negatively but also exacerbates emotional well-being issues due to the lack of access to resources and the stigma surrounding menstruation. Studies have shown that individuals who menstruate report higher levels of distress, anxiety, and depression because of these challenges [3,6,24-26].

Research examining the incidence of period poverty and its effects on the mental health of college-aged women in the United States found that numerous young women are unable to afford necessary menstrual products each month, which could affect their mental health [25]. Supporting these young women is essential for better access to cost-effective menstrual products. The stigmatization of menstruation exacerbates the impact of period poverty, leading to psychological distress and social ostracism. Societal taboos and myths surrounding menstruation persist in many cultures, fostering an environment of silence and shame. In Malawi, for example, menstruating girls are often considered impure and are excluded from communal activities, contributing to feelings of isolation and low self-esteem [26]. Similarly, in Nepal, despite legal prohibitions, the practice of Chhaupadi, which entails sequestering menstruating women in makeshift huts, still endures in some communities [27]. In some regions of Afghanistan, cultural views on infertility inhibit menstruators from touching or cleaning their genital areas, which heightens their risk of urogenital infections [28]. This stigmatization not only impacts mental health but also exacerbates gender inequalities by reinforcing the subordination of women and limiting their participation in social, educational, and economic activities [29].

Economic vulnerability is intrinsically linked to period poverty. The high cost of menstrual products relative to income in many low- and middle-income countries significantly burdens strained household budgets. According to research conducted in the slums of Dhaka, Bangladesh's capital, due to a restricted budget, nearly 95% of women and 90% of adolescent girls reused rags during menstruation without proper cleaning. This practice led to scabies in the vaginal area, urinary infections, and pregnancy complications. Additionally, the use of low-quality, reused clothes often dyed with harmful substances likely increases the risk of various urogenital diseases for women [30,31]. Even in higher-income countries, period poverty remains a relevant issue. In the United Kingdom, a survey conducted by Plan International found that one in ten girls could not afford menstrual products at some point, highlighting that this issue transcends economic boundaries [32]. The financial stress imposed by the need for menstrual products can force individuals to trade for other essential needs, such as food or education, compounding the cycle of poverty [32,33].

In addition to personal hardships, period poverty has broader economic implications. The loss of productivity due to absenteeism from work or school leads to significant financial costs. A report by the World Bank [34] estimated that inadequate menstrual health management results in global economic losses of billions of dollars annually due to reduced productivity and educational attainment [35]. Therefore, addressing period poverty is a matter of equity, human rights, and an economically prudent strategy that can yield substantial benefits for societies.

The nexus between period poverty and sustainable development goals

**Figure 4:**
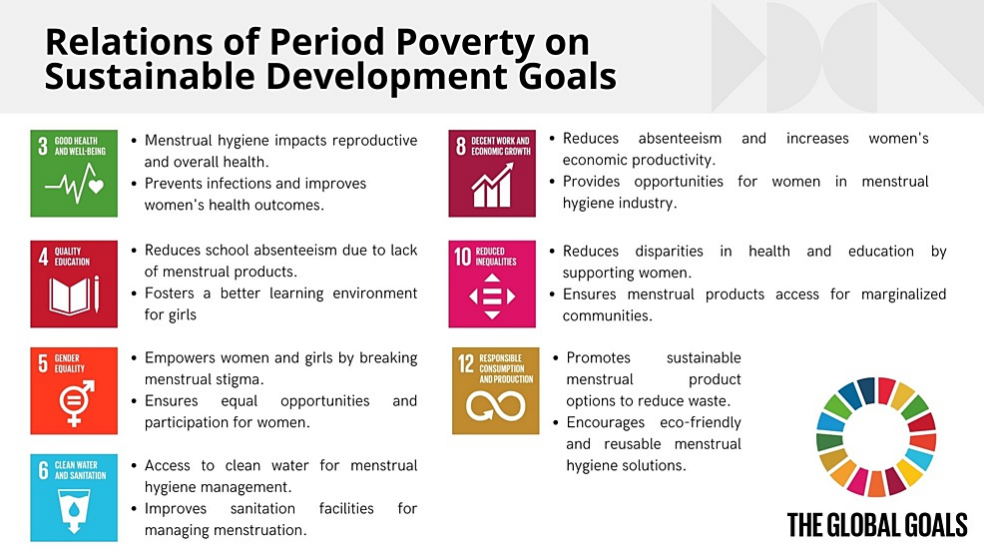
Relations of period poverty on Sustainable Development Goals. Image Credit: Nor Faiza Mohd. Tohit.

Combating period poverty is essential for advancing several SDGs outlined by the United Nations, which aim to address global challenges and improve lives worldwide by 2030. Period poverty is a lack of access to menstrual products, education, sanitation facilities, and waste management. Various aspects of well-being and development intersect with multiple SDGs, including health, education, gender equality, and economic growth [9-11]. Figure 4 illustrates the relationship between period poverty and SDGs.

Tackling Period Poverty Is Integral to Achieving SDG 3: Good Health and Well-Being

Menstrual health is a critical aspect of overall health. The absence of sanitary products forces many to resort to unhygienic alternatives, increasing the risk of infections such as urinary tract infections and reproductive health issues [30,31]. Poor menstrual hygiene management can also lead to severe medical conditions and mental health problems, including stress and anxiety exacerbated by shame and stigma [25,26]. A study highlights that improving menstrual hygiene management is crucial for preventing health complications and promoting well-being among women and girls [35]. Ensuring access to sanitary products and proper menstrual hygiene education can significantly improve health outcomes and enhance quality of life.

Addressing Period Poverty Is Fundamental to SDG 4: Quality Education

Across many developing countries, period poverty contributes to high rates of school absenteeism among girls, jeopardizing their educational opportunities and prospects. In India, inadequate sanitation facilities and menstrual stigma contribute to dropout rates among adolescent girls [19]. By providing accessible menstrual products and ensuring adequate sanitation facilities in schools, we can significantly reduce absenteeism, ensuring that girls receive an uninterrupted education. Education policies integrating menstrual health management can empower girls to stay in school and complete their education, thereby breaking the cycle of poverty and dependency [16-20].

Period Poverty Also Critically Intersects With SDG 5: Gender Equality

Menstruation-related stigma and cultural taboos reinforce gender inequalities, limiting the participation of women and girls in societal activities. In many cultures, menstruating women and girls are often seen as impure or unclean, which can lead to practices that marginalize them [27]. Combating period poverty involves not only providing menstrual products but also challenging and changing the cultural attitudes that perpetuate these stigmas. Educational initiatives that promote menstrual health awareness and de-stigmatization can significantly contribute to gender equality by empowering women to participate fully in societal, academic, and economic activities without the constraints of period-related stigma [23,24,26].

SDG 6

Clean water and sanitation are closely related to menstrual hygiene management. Many women and girls lack access to clean water, private sanitation facilities, and proper waste disposal systems, making it challenging to manage menstruation safely and with dignity [36]. Lack of appropriate facilities often forces girls to miss school or women to stay home from work, highlighting the importance of integrating menstrual hygiene management into water, sanitation, and hygiene (WASH) programs [31,33]. Improved WASH infrastructure in schools, workplaces, and public places, which includes clean water, private sanitary spaces, and adequate waste disposal, is crucial for ensuring the health and hygiene of menstruating individuals [35].

Period Poverty Also Has Significant Implications for SDG 8: Decent Work and Economic Growth

The lack of access to menstrual products reduces workforce participation and productivity, as many women and girls may miss work during their menstrual cycles. A report by the World Bank estimated that inadequate menstrual health management leads to significant economic losses due to reduced productivity and diminished educational outcomes [34]. By ensuring that menstrual products and hygiene facilities are accessible and affordable, we can enhance workforce participation and productivity, which contributes to economic growth [37]. Efforts to promote menstrual health in the workplace, such as providing free menstrual products and creating supportive environments, are vital for fostering inclusive economic development.

Addressing Period Poverty Also Aligns With SDG 10: Reduced Inequalities

Marginalized communities, including those living in poverty, in rural areas, and among refugees, often face the brunt of period poverty [28]. The lack of access to menstrual products and services exacerbates existing social and economic inequalities, preventing these communities from accessing essential opportunities [29]. Targeted interventions that provide menstrual products and education to these vulnerable groups are critical for reducing inequalities and ensuring that all women and girls can manage their menstruation with dignity [38].

SDG 12

Responsible consumption and production emphasize the importance of sustainable menstrual waste management. Traditional disposable menstrual products contribute to significant environmental pollution, with millions of used sanitary pads and tampons in landfills annually [39]. Promoting sustainable menstrual products, such as reusable cloth pads or menstrual cups, is aligned with reducing waste and promoting sustainable consumption practices [40]. Educational campaigns encouraging sustainable menstrual products can mitigate environmental impacts while addressing the hygiene needs of menstruating individuals [41].

Combating period poverty as a multifaceted approach

Efforts to combat period poverty are gaining momentum, with increasing advocacy and policy interventions aimed at improving menstrual hygiene management. Countries like Scotland have taken pioneering steps by providing free period products to all who need them, setting a benchmark for other nations [42]. Similar initiatives are being implemented in various parts of the world. In New Zealand, the government announced a policy to provide free sanitary products in schools, targeting period poverty and its impact on education [43]. These policies not only provide immediate relief but also contribute to breaking the stigma and normalizing menstruation as a natural aspect of life.

Educational campaigns are fundamental to addressing period poverty and breaking down associated stigmas. Organizations such as WaterAid are actively engaged in community-based education programs that teach about menstrual health management. In Nepal, WaterAid's comprehensive program provides menstrual products and education on proper usage and reproductive health, leading to improved menstrual hygiene practices and reduced absenteeism [44]. Integrating menstrual health education into school curricula can foster a more informed and accepting generation, reducing the stigma associated with menstruation and promoting gender equity [38,39].

Infrastructure improvements are also crucial to addressing period poverty. Ensuring access to clean water, private sanitation facilities, and proper waste disposal systems is essential for effective menstrual hygiene management [45]. In India, the Swachh Bharat (Clean India) Mission has significantly improved sanitation infrastructure [46]. However, targeted efforts are needed to ensure that these improvements address the specific needs of menstruating women and girls. Inadequate infrastructure hampers menstrual hygiene and contributes to broader public health issues, such as urinary tract infections and other sanitation-related diseases [47-50].

It is imperative to enhance the training of health workers regarding menstrual health, menstrual disorders, and approaches that are sensitive to gender in addressing the needs of everyone who menstruates, such as girls, women, individuals with disabilities, transgender individuals, and non-binary people. Our healthcare systems must recognize menstruation as a critical indicator of health and well-being and a vital measure of population health [51].

Non-governmental organizations and grassroots movements are pivotal in filling the gaps left by government initiatives. Numerous resources delve into the efforts of NGOs to combat period poverty. One example is an article acknowledging the role of NGOs in menstrual activism [52]. Another source discusses the impact and initiatives of NGOs in menstrual hygiene management [53]. These works offer insights into NGOs' strategies and programs to address menstrual hygiene challenges and alleviate period poverty among marginalized communities. The Red Dot Foundation's #HappyPeriod campaign in India focuses on breaking menstrual taboos through dialogue and education, reaching thousands of women and girls in rural and urban settings [54]. Similarly, in Malaysia, initiatives like the "Bunga Project" aim to provide sanitary products to underprivileged women and girls alongside educational programs to raise awareness about menstrual health [55].

Research is essential to understand the multifaceted nature of period poverty and to develop effective interventions. Recent studies have highlighted the interconnectedness of period poverty with broader issues such as gender-based violence, economic inequality, and health disparities [56-58]. Research in Kenya revealed that high rates of sexual and reproductive health (SRH) harm and interrupted schooling are global challenges for adolescent girls, requiring effective interventions [58]. The impact of menstrual cups or cash transfers conditioned on school attendance, or both, on SRH and schooling outcomes was assessed. Understanding these complex dynamics is crucial for developing holistic and sustainable solutions to period poverty.

Research on period poverty should encompass studies examining the socio-economic impact, effectiveness of policy interventions, and innovative menstrual hygiene solutions. For instance, recent works such as Plesons et al. shed light on the need for comprehensive research to inform effective interventions [59]. A systematic review [60] also offers valuable insights into addressing period poverty through education, access, and policy change. Addressing period poverty requires a multifaceted approach that involves cross-sector collaboration. Government agencies, non-governmental organizations, private sector entities, and community leaders must work together to develop and implement comprehensive strategies that address the root causes and manifestations of period poverty. Policies should be informed by evidence-based research and should prioritize the needs and voices of those affected by period poverty. Table 1 summarizes the key stakeholders and their roles in combating period poverty.

**Table 1:** The key stakeholders and their roles in combating period poverty.

	Example	Roles
Coordinating body		
Government or major NGO	Ministry of Health, the United Nations, and a prominent NGO like Plan International.	Leads and oversees efforts to address period poverty.
Key stakeholder categories		
1. Government agencies	Ministries of Education, Health, and Women's Affairs.	Policy formulation, funding, and infrastructure development.
2. Non-governmental organizations (NGOs)	Local and international NGOs working on health, education, and gender equality.	Program implementation, community outreach, and advocacy.
3. Educational institutions	Schools, universities, and teacher training institutes.	Menstrual hygiene education, distribution of products, and creating gender-sensitive environments.
4. Healthcare providers	Hospitals, clinics, and healthcare professionals.	Providing menstrual health services information dissemination.
5. Private sector	Companies producing menstrual products have corporate social responsibility (CSR) initiatives.	Product innovation, distribution, funding, and advocacy campaigns.
6. Communities and local leaders	Community groups, religious leaders, and local councils.	Cultural change, community-level education, and stigma reduction.
7. International organizations	UNICEF, WHO, UNESCO.	Global advocacy, funding, policy guidance, research.
8. Media and communication platforms	News media, social media influencers.	Raising awareness and advocacy through storytelling and campaigns.
Specific collaborations and joint initiatives		
1. Public-Private Partnerships (PPP)	Collaboration between government agencies and the private sector for funding and distribution of menstrual products.	The government contracts with companies to supply schools with menstrual products.
2. NGO and Community Groups Collaboration	Local NGOs were working with community leaders to conduct menstrual health workshops.	Conducting community-based workshops to educate about menstrual hygiene.
3. Education and Health Sector Partnership	Joint efforts of educational institutions and healthcare providers to offer comprehensive menstrual health education and services in schools.	School health programs integrate menstrual hygiene management into their curriculum.
4. International and Government Agency Collaboration	International organizations support national governments with technical and financial aid.	UNICEF is working with the Ministry of Education to improve menstrual hygiene facilities in schools.

Limitation of the study

While the study on period poverty provides valuable insights, several limitations should be noted. First, the research may be bounded by data availability and quality, particularly in marginalized communities where access to research resources and information may be limited [61]. This could impact the representativeness of the findings and the ability to generalize the results to broader populations. Additionally, the study's reliance on self-reported data from individuals affected by period poverty may introduce response bias or underreporting due to the topic's sensitive nature [62,63].

Furthermore, the study's scope may be constrained by its geographical focus, potentially overlooking variations in the experiences of period poverty across different cultural and socio-economic contexts. The lack of longitudinal data and limited long-term follow-up could also hinder a comprehensive understanding of the persistent impact of period poverty on individuals' lives.

Lastly, the study may not fully capture the complex interplay of factors contributing to period poverty, such as policy dynamics, cultural norms, and economic disparities, necessitating further in-depth exploration. Acknowledging these limitations is crucial for contextualizing the study's findings and guiding future research and policy efforts to address period poverty.

Future recommendation for research

Future research on period poverty should focus on several key areas to further deepen our understanding of this critical issue. First, there is a compelling need for longitudinal studies to assess the long-term impact of period poverty on individuals' health, education, and economic outcomes. Understanding the lasting effects of inadequate menstrual hygiene management is crucial for developing effective interventions and policies. Additionally, research should explore the intersectionality of period poverty, considering the varying impact on different communities based on factors such as race, socioeconomic status, and geographic location. Such nuanced investigations can help tailor interventions to specific needs and mitigate disparities.

Further exploration into the underlying causes of period poverty, including socio-cultural, economic, and policy-related factors, is essential for informing targeted and sustainable solutions. Studies should also examine the effectiveness of existing interventions and programs to address period poverty, focusing on identifying best practices and scalable models. This encompasses evaluations of distribution programs, education initiatives, and advocacy efforts to understand their impact and potential for replication in diverse contexts.

In addition, future research must emphasize the voices and experiences of individuals affected by period poverty, ensuring that their perspectives are central to designing and evaluating interventions. Community-based participatory research can provide valuable insights and foster the co-creation of culturally and contextually relevant solutions.

Furthermore, given the global nature of period poverty, comparative research across different regions and countries can yield valuable lessons and facilitate the exchange of best practices. Finally, research focusing on the policy landscape surrounding menstrual hygiene management and period poverty is vital, aiming to assess the incorporation of menstrual health into broader public health and development policies and frameworks.

By prioritizing these areas in future research endeavors, we can advance our knowledge of period poverty, strengthen evidence-based interventions, and work towards ensuring menstrual health and dignity for all individuals, aligned with the targets of sustainable development, gender equality, and public health agendas worldwide.

## Conclusions

In conclusion, period poverty is a pressing public health issue with far-reaching implications for health, education, and economic well-being. It exacerbates existing inequalities and poses significant barriers to achieving gender equity. Addressing period poverty requires a concerted effort to provide access to affordable menstrual products, improve sanitation infrastructure, enhance educational efforts, and implement supportive policies. It is essential to challenge and dismantle the cultural stigmas associated with menstruation and to promote a more informed and equitable society. The examples from around the world, including Malaysia, demonstrate the challenges and the potential for positive change. By prioritizing menstrual health as a critical component of public health, societies can create an environment where all individuals can manage their menstruation with dignity and achieve their full potential.
